# Changing nursing practice within primary health care innovations: the case of advanced access model

**DOI:** 10.1186/s12912-020-00504-z

**Published:** 2020-12-02

**Authors:** Sabina Abou Malham, Mylaine Breton, Nassera Touati, Lara Maillet, Arnaud Duhoux, Isabelle Gaboury

**Affiliations:** 1 School of Nursing, Faculty of Medicine and Health Sciences, Université de Sherbrooke, Longueuil, Québec Canada; 2 Charles Lemoyne– Saguenay–Lac-Saint-Jean sur les innovations en santé (CR-CSIS) Research Centre, Campus Longueuil, 150 Place Charles-Lemoyne, Room 200, Longueuil, Québec J4K 0A8 Canada; 3 Department of Community Health Sciences, Faculty of Medicine and Health Sciences Université de Sherbrooke, Sherbrooke, Québec Canada; 4 Canada Research Chair - Clinical Governance in Primary Health Care (Tier 2), Sherbrooke, Québec Canada; 5 École Nationale d’Administration Publique, 4750 avenue Henri-Julien, 5th floorl, Montréa, Québec H2T 3E5 Canada; 6 Faculty of Nursing, Université de Montréal, Montréal, Québec H3C 3J7 Canada; 7 Department of Family Medicine and Emergency Medicine, Faculty of Medicine and Health Sciences, Université de Sherbrooke, Sherbrooke, Québec Canada

**Keywords:** Advanced access, Primary health care, Nurses practice change, Nurse optimization

## Abstract

**Background:**

The advanced access (AA) model has attracted much interest across Canada and worldwide as a means of ensuring timely access to health care. While nurses contribute significantly to improving access in primary healthcare, little is known about the practice changes involved in this innovative model. This study explores the experience of nurse practitioners and registered nurses with implementation of the AA model, and identifies factors that facilitate or impede change.

**Methods:**

We used a longitudinal qualitative approach, nested within a multiple case study conducted in four university family medicine groups in Quebec that were early adopters of AA. We conducted semi-structured interviews with two types of purposively selected nurses: nurse practitioners (NPs) (*n* = 6) and registered nurses (RNs) (*n* = 5). Each nurse was interviewed twice over a 14-month period. One NP was replaced by another during the second interviews. Data were analyzed using thematic analysis based on two principles of AA and the Niezen & Mathijssen Network Model (2014).

**Results:**

Over time, RNs were not able to review the appointment system according to the AA philosophy. Half of NPs managed to operate according to AA. Regarding collaborative practice, RNs were still struggling to participate in team-based care. NPs were providing independent and collaborative patient care in both consultative and joint practice, and were assuming leadership in managing patients with acute and chronic diseases. Thematic analysis revealed influential factors at the institutional, organizational, professional, individual and patient level, which acted mainly as facilitators for NPs and barriers for RNs. These factors were: 1) policy and legislation*;* 2) organizational policy support (*leadership and strategies to support nurses’ practice change);* facility and employment arrangements (*supply and availability of human resources);* Inter-professional collegiality; 3) professional boundaries; 4) knowledge and capabilities; and 5) patient perceptions.

**Conclusions:**

Our findings suggest that healthcare decision-makers and organizations need to redefine the boundaries of each category of nursing practice within AA, and create an optimal professional and organizational context that supports practice transformation. They highlight the need to structure teamwork efficiently, and integrate and maximize nurses’ capacities within the team throughout AA implementation in order to reduce waiting times.

## Background

Transforming nursing practice in primary healthcare settings is recognized as a promising strategy for improving the quality and efficiency of primary care and addressing the unmet healthcare needs of individuals, families, and communities [1, 2]. Scholars worldwide highlight the prominent role nurses can play in shifting to new models of care to improve primary healthcare services [2, 3]. A large and solid body of evidence emphasizes the central role of nurses in improving the accessibility of primary care services, the quality of care, and the satisfaction of both patients and other professionals. Two recent systematic reviews show that, compared to primary care physicians, trained nurses (e.g. nurse practitioners [NPs], registered nurses [RNs]) offer equal or better quality of care, and likely achieve equal health outcomes [4] and higher levels of patient satisfaction with regard to urgent physical problems and chronic conditions [3]. More specifically, Contandriopoulos, Brousselle [5] reveal how introducing NPs into primary care teams contributes to disrupting the status quo and reforming primary care delivery models.

However, despite the essential and strategic role nurses play in transforming primary care through reorganization and/or innovation [2], nursing practice has received limited attention in research on innovative models aiming to reduce wait times for primary care appointments, such as advanced access (AA). The current state of knowledge does not enable us to understand changes in nursing practice required in these models nor the challenges they present.

Advanced access is a widely recognized innovative model designed to improve timely access to primary care services that respond to patients’ needs and preferences on time [4, 6]. It builds on five guiding principles (Table 1) [7]: 1) balancing supply and demand, 2) reducing work backlog (i.e. eliminating wait lists), 3) reviewing the appointment system, 4) developing/improving inter-professional collaborative practice, and 5) developing contingency plans. In AA, appointments are offered regardless of the reason for the visit and the urgency of the need [7, 8].

**Table 1:** The key principles of advanced access, adapted from Murray & Berwick (2003) [6], Breton et al. (2017) [7]

Key principles of Advanced Access	Definition
*1 Balance supply and demand*	To assess and understand, on the one hand, the actual patient demand for appointments per physician/health professional per day, weighted by the patients’ status and, on the other hand, the supply (e.g., number of appointments offered), in order to achieve the right balance between the two, matching demand with supply. Strategies to decrease demand for visits (e.g., max pack, extending visit intervals) or to increase supply (e.g., redesigning doctor/health professional scheduling systems) are used.
*2 Reduce the backlog of previously scheduled appointments*	To eliminate previously scheduled appointments (wait list) through many strategies, such as adding resources or increasing the supply of visits during a period of time. A communication strategy must also be put in place to inform and educate patients about the new advanced access model.
*3 Review the appointment system*	To plan physicians’ (health professionals such as nurses) schedules over a short term (two to four weeks) and smooth out the demand for visits in order to offer same-day appointments for acute and urgent cases or quick appointments according to patients’ needs.
*4 Integrate inter-professional practice*	To develop or enhance inter-professional collaborative practice between physicians and other healthcare providers (e.g., nurses). Professional roles need to be optimized and tasks need to be clarified to respond to patient needs in a timely manner.
*5 Develop contingency plans*	To plan for seasonal increases in demand and to develop coverage plans for replacing medical staff or other healthcare providers during vacation and sick leave. Many strategies are applied, such as increasing the number of slots prior to leave and after returning to duty, hiring temporary providers, and distributing and matching staffing competencies to demand. Integrating collaborative and interdisciplinary practice facilitates planning for periods of absence.

Implementation of the AA model requires reorganizing the practice of all team members [9, 10], including various categories of nurses who have to adapt their tasks and practice to ensure that implementation is effective and patient-focused [11, 12]. Indeed, optimizing the role of nurses is a key strategy for improving timely access, and meeting health needs and demands [11]. For instance, nurses can reduce the number of prescheduled appointments and open up more appointment slots for same or next day appointments, in order to respond to demand, reduce delays, and restore the balance required for the model to be successful [7]. They can reschedule visits to manage uncomplicated acute conditions/illnesses and free up physicians to see more complex patients, thus increasing care team capacity and reducing wait times for appointments [13]. Nurses can also, through enhanced collaborative practice with family physicians and other providers, manage patients with chronic conditions and thus reduce the number of physician visits.

While there is abundant literature investigating family physician (FP) practice change to implement key principles of AA and measure outcome indicators (e.g. reductions in wait times and missed appointments), less research is available on how changes in each category of nurses’ practice with regards to appointment rescheduling, are achieved within this model. Moreover, the inter-professional aspect of AA (4th principle), which emphasizes enhanced collaborative practice – between RNs, NPs, physicians, and clerical staff – to maximize the efficiency and quality of care, has not yet received much research attention.

A few studies show that changing nursing practice (e.g., taking less complex tasks from physicians) has contributed to the implementation success of AA. However, these studies rarely address how the transformation took place and whether it applies to a particular nurse category. For example, two studies [14, 15] identify use of NPs and RNs as a way to increase practice efficiency in AA, but do not specify how each type of nurse’s practice was optimized within AA – in terms of appointment rescheduling and interprofessional collaboration – nor identify success factors or draw lessons that could be extended to other contexts. Some studies conducted in Québec show that optimizing RN practice by including patient management and pregnancy follow-up in their practice enhances collaborative practice. Nonetheless, little is known about what collaborative models and key factors lead to successful nursing practice change [7, 16].

The one review of team experience with AA, conducted in a newly established nurse practitioner -led clinic in northern Ontario [17], underscores benefits such as increased NP control over their work day, greater patient satisfaction and safety, and decreased walk-in and emergency room visits. The study also points to challenges in terms of unmet client expectations when there is a mismatch between supply and demand, continued need to triage calls and skills development, and the need for flexibility in adapting operational processes. The review calls for further research to explore challenges around nurses’ practice that emerge within AA. This call was reiterated in a recent evidence synthesis [18], which highlights large knowledge gaps with regards to strategies used by various stakeholders to implement the model. Given that RNs and NPs have considerable responsibility for bringing about patient centred primary care, the present study aims to understand the changes involved within each of their practice. Results will help to guide reorganization of care processes and distribution of work to enhance healthcare team capacities and point to ways nursing practice can be adjusted to improve timely access to primary care.

This study is the first to report on NP and RN experience of practice change following implementation of the AA model.

Its main objectives of are to explore nurse practitioners’ and registered nurses’ experiences of practice changes throughout the implementation of the AA model and identify factors that facilitate or impede change.

The specific research objectives are to:

1. analyze RN and NP practice change with regard to appointment scheduling in AA;
2. describe changes in collaborative practice between RNs, NPs and physicians within the AA model.
3. Identify contextual factors that influence changes in nursing practice with AA.

### Conceptual framework

The study employs a framework that builds on the principles of AA elaborated by Murray and Tantau (2000) [8] and on the “networked model” developed by Niezen & Mathijssen (2014) [19]. The Murray and Tantau (2000) model is used to operationalize nursing practice change with respect to two principles of AA: appointment scheduling by nurses working or attempting to work according to the AA model (3rd principle), and inter-professional collaborative practice between nurses and FPs (4th principle). Niezen & Mathijssen’s “networked model” stipulates that adapting to new realities (in this case the AA model) requires changes in nursing practice and a reallocation of tasks among different professional groups. It serves as a conceptual lens to understand the dynamic interaction between four levels of environment, representing the context within which change takes place, that influence change in nursing practice within healthcare systems. These include the *internal environment* – (1) characteristics of nurses (e.g., knowledge and capabilities); (2) professional boundaries (e.g., physician-nurse collaboration, trust, physician job security, etc.) –, and the *external environment*: (3) the organizational environment (e.g., organizational policy support, facility and employment arrangements, type of health setting, inter-professional collegiality); and (4) the institutional environment (e.g., legislation, government policies, socioeconomic forces).

Given that AA is a patient-centered model, and is designed to respond to the needs and preferences of patients [11], we do not consider patients as part of the external environment, but rather as a separate influence, placed at centre of our conceptual model.

## Methods

### Research setting

Improving timely access to primary health care is among the main objectives of the Ministry of Health and Social Services (MHSS) in Quebec. AA is promoted by many professional medical associations – the College of Family Physicians (CQMF) and the Federation of General Practitioners (FMOQ) – in Québec, and adoption of the model has expanded exponentially since 2012. Widespread implementation of AA is a Ministry priority and figures as a formal target in the management framework introduced in 2015 for all university family medicine groups (UFMGs) [20]. To this end, FPs receive financial incentives for attending training sessions [21]; training has been provided to over 2000 healthcare providers, including RNs, NPs and clerical staff. These concerted efforts to implement the AA model in the majority of primary health care (PHC) clinics across Quebec provide a privileged context for analyzing nursing practice change in AA.

### Research design and data collection

This study is nested within a larger multiple case study conducted in four UFMGs in the province of Quebec [16] that are considered early adopters of AA. University Family medicine groups (UFMGs) are public primary healthcare organizations with a teaching mission, devoted to training family medicine residents [22], and enabling them to learn how to work as part of an inter-professional team including FPs, nurses, pharmacists and social workers. Selection of the four FMUs is based on two criteria: 1) having at least 1 year of experience in implementing AA; and 2) representing diverse socio-demographic environments (rural and urban UFMGs). The characteristics of each UFMG are shown in Table 2.

**Table 2:** Characteristics of the selected University Family medicine groups

Setting	UFMG 1	UFMG2	UFMG3	UFMG4
	Urban	Urban	Urban	Rural
**Team composition**				
Family physicians	33	20	13	15
Residents 1st, 2nd year (R1-R2)	25	24	13	14
Advanced practice nurse	2	1	1	1
Registered nurse	4	4	1	2
Clerical staff	4	2	2	4
**Registered patients**	11,000	10,000	< 6000	6700
**Patient population served**	All types, ages (Pediatric, pregnant women, young families, elderly, vulnerable patients, etc.)	All types, ages	All types, ages	All types, ages

*UFMG* University Family medicine group, *IUHSSC* Integrated university health and social services center; R1 = First year of residency; R2 = Second year of residency

We adopt a longitudinal qualitative research design using a recurrent cross-sectional approach [23] to examine nurse practice change. We aim to capture changes in nurses’ perceptions at two different time points following the implementation of AA. The sample consists of 11 participants (a sub-sample of respondents in the multiple case study), including two types of nurse: NPs (*n* = 6) and RNs (*n* = 5). Nurse practitioners (NPs) are registered nurses with a Master’s degree who possess and demonstrate competencies to diagnose, with supervision of a FP, six chronic diseases, as well as order and interpret some diagnostic tests, prescribe medication and medical treatment, and perform specific procedures within their legislated scope of practice and in collaboration with at least one physician [24]. Their activities cover health promotion, preventive care, treatment and follow-up for common acute health problems, follow-up during pregnancy, and monitoring and management of chronic diseases [25]. Registered nurses (RNs) hold a baccalaureate degree in nursing. Their key activities in primary care include managing chronic diseases, supporting physicians in clinical activities, providing patient education, counselling and self-care management support, applying collective orders to perform clinical activities (e.g., administering or adjusting medication, etc.), assuring systematic and joint follow-up of patients, and participating in decision making [26].

Within unit teams, nurses were purposively selected based on their involvement in implementing the AA model, or the extent to which their practice was likely to be impacted by AA implementation. Participants were informed of the study aim and were invited by email to participate in interviews. All accepted and were interviewed twice, except one participant (NP) who did not reply to two invitations for the follow-up interview and was replaced by another NP for the second interview resulting in a total of 6 NPs interviewed. Nurses were interviewed twice: in January 2016 (more than 1 year after implementation began), and again 14 to 18 months later (between May and August 2017). Three researchers (SAM, MB, LM) undertook the first interviews and one researcher (SAM) conducted the follow-up interviews using a semi-structured interview guide.

Questions from the interview guide included: “How has your nursing practice been transformed since the implementation of advanced access?” Please describe your experience in making this change:“How the planning of your time slots changed following the implementation of this model? How and which changes were made to your schedule and the nature of your consultations”; “How did your collaboration with other healthcare professionals (e.g., physicians, etc.) change? Could you describe this collaborative practice change?” “How do you think the context has influenced changes in nurse practice within this model?” [see Additional file 1].

Contextual factors were addressed during the second interview to understand why changes in nursing practice in AA did or did not occur.

Twenty interviews were conducted in total, each lasting between 45 and 60 min. All were conducted in French at the participants’ workplace, except two interviews conducted by telephone with participants located over 150 km away from the research centre. Interviews were audio-recorded and transcribed verbatim. Field notes were kept to reflect on changes (e.g., to appointment systems and collaborative practice) over time. Demographic information was also collected and included the age, sex, education, and years of experience of participants. All participants signed an informed consent form prior to the study. The study was approved by the Research Ethics Board of the Centre Intégré Universitaire de Santé et de Services Sociaux de l’Estrie – Centre Hospitalier Universitaire de Sherbrooke.

### Data analysis

We used a deductive thematic approach to analysis while remaining open to emerging themes. Data analysis involved several steps. Interview transcripts were read and reread. A narrative case history of seven to eight pages was created for each time point for each participant. They were examined to get a sense of the global change within each FMU. All transcribed interviews were coded manually and via QDA Miner version 4.0 using a coding grid based on the study’s conceptual framework. The coding grid covered the following themes for each group of nurses: scheduling nursing appointments, models of collaborative practice, and factors (institutional, organizational, professional, individual, patient) influencing nursing practice changes within AA.

Data were summarized using matrices following two steps. They were sorted by theme and subtheme with illustrative quotes, and entered into a matrix based on the coding grid and the two time periods [23]. Separate matrices were first prepared for each participant within a same FMU and summarized within one matrix (individual matrices). Themes and subthemes from each matrix were then reorganized into a new analytical matrix to compare data sets from interview 1 and interview 2 and identify changes across time (longitudinal matrices) [23]. Individual and longitudinal matrices were examined by three authors (SAM, MB, IG). Different methods were used to enhance the trustworthiness of data: findings were discussed among the research team to confirm findings; a summary of results was shared with nurses in the four FMUs under study; and an audit trail was kept to describe steps in the analysis. Nurse feedback on study findings was taken into consideration and integrated into the final results.

## Results

A summary of participant characteristics is presented in Table 3. All RNs were female, ranging in age between 37 to 58 years old, with a mean 24 years of work experience. All held a Bachelor’s degree in nursing sciences. The mean age of NPs was 32 years (range 28–38 years) with a mean 5 years work experience. They all held a Master’s degree in nursing science and a postgraduate degree in advanced nursing practice with a specialization in primary care.

**Table 3:** Characteristics of nurses

Characteristics	Job title	
	Nurse practitioner (*N* = 6)	Registered nurse (*N* = 5)
**Age (n, %)**		
20–30	4 (66.66)	0
31–40	2 (33.33)	2 (40)
41–50	0	2 (40)
> 51	0	1 (20)
**Sex (n)**		
Female	5 (83.33)	5 (100)
Male	1 (16.66)	0
**Educational level (n, %)**		
Nursing college diploma	0	0
Bachelor degree	6 (100)	5 (100)
Master’s degree	6 (100)	0
Postgraduate degree in advanced nursing practice	6 (100)	0
**Years of professional experience**		
Mean	5	24
Range	0,5–11	19–35
**Years of professional experience (n, %)**		
< 5	4 (66.66)	0
5–10	1 (16.66)	0
11–15	1 (16.66)	0
16–20	0	2 (40)
21–25	0	2 (40)
≥ 26	0	1 (20)

We present findings on practice change for each category of nurse with regards to: (1) appointment-scheduling models, (2) inter-professional collaborative practice, and (3) factors influencing changes in nursing practice.

1. **Appointment-scheduling models**

AA relies on balancing capacity to provide appointments with demand for appointments. For NPs and RNs, this means that appointment supply meets demand and therefore involves nursing practice gaining capacity by reviewing their appointment schedules to accommodate patient demand for visits and meet their needs.

### Nurse practitioners

Our data show that NPs were still not all making appointments based on in-depth analysis of demand and supply. However, some NPs in our study took steps to measure demand profiles and reduce the imbalance in supply and demand either on regular basis or occasionally.

"Since coming back [from long-term leave], no, I haven't performed the exercise [evaluated supply and demand]. I did it initially, I did it before I went on leave as well because I had to decide to whom I would assign the follow-ups". (NP5-Interview 2)

With regards to redesigning the appointment system, some NPs adopted and maintained the same AA scheduling template over time: 80 to 90% of appointment slots were left open over a two-to-3 week period; and 10 to 20% were pre-booked for patients unable to call back (e.g. elderly patients, patients with cognitive impairments). It is noteworthy that few NPs managed to make open slots available over time to accommodate all types of patient needs (e.g., sore throat, follow-up of a chronic disease, etc.) without making any distinction between appointment types. Instead of triaging patients by type of appointment (urgent or follow-up visits), clerical staff sorted appointment demand by healthcare provider to reduce scheduling complexity, delays and time spent on the phone.

"It's true that there has been some change. At first, we had rapid appointment slots and follow-up appointment slots. We eliminated that because it was complex for the clerical staff. Sometimes they called me, "Can you see Mrs. X for a follow-up of diabetes in a rapid slot appointment", which became very complicated. What I'm doing now is trying to get more supply than demand and all my 30-minute time slots are available for either a follow-up appointment, a pap test, an urgent appointment, whatever, access is there. "(NP1- Interview 2)

Other NPs did not manage to gain capacity in their schedules and operate in “*real AA”*. They maintained over time a predominantly pre-booked appointment model over a two- to three-month period, with two open slots each day reserved to respond to urgent care needs and minor illnesses.

### Registered nurses

At the initial interview, most RNs reported having made some change to the appointment system. This scheduling change represented a shift from a traditional appointment system, with all daily appointments pre-booked two to 3 months in advance, to increased availability by keeping two or three slots per day open to accommodate patients with urgent healthcare needs or minor conditions/illnesses and episodic complaints (e.g. distressed patients, sore throat, fever and cold, symptoms of urinary tract infection, etc.).

"My appointment schedules are not over two or three weeks. I really wanted to have a schedule over a two-month period because, as I manage it by myself, I know what I put into it. It is not open booking on the long term, but I am able to cope with emergencies.

How many slots a day?

Two late afternoon slots easily. Then in the morning, I often get one. I’m able to take at least three emergencies, or patients who need to be seen quickly, a day" (RN3--Interview 2).

However, over time, some RNs had to go back to the traditional pre-booked appointment system, following the introduction of additional NPs in the unit, and the redirection of patients with minor acute illnesses (e.g., symptoms of urinary tract infection, ear infections, etc.) from RNs to the NPs.

"That's the most important change. So, I think that AA as such has significantly increased accessibility to the physician; but at the same time, the fact that nurses also operate in advanced access, we have fast open slots almost every day and are more able to meet the particular needs of patients" (RN1--Interview 1).

“It’s almost 100 percent planned in advance now [...] That’s what I’m saying, the advanced access does not include us anymore […]. It has changed, I would say, because of the availability of family physicians and the introduction of new staff [NPs] in the unit” (RN2--Interview 2).

Over the study period, other RNs maintained a mixed system of predominantly pre-booked appointments scheduled eight to 12 weeks in advance and a few open slots for unscheduled care (same-day appointments). Some nurses referred to these open slots as their “AA appointments” and kept them for patients who needed to be seen urgently after calling from home or being referred by FPs working in collaborative practice with the RNs. Pre-booked appointments were mainly for follow-up visits (e.g., chronic care patients), and management of new patients. Nurses expressed concerns with reducing pre-booked appointments, particularly for older people, patients with chronic illness, and patients on blood thinners (Coumadin regimens) who require consistent management. Nurses felt that these pre-booked appointments secured access for patients with complex medical problems or cognitive impairments who required close follow-up. Nevertheless, RNs mentioned some benefits of open appointments, including the ability to handle pressing patient concerns and urgent conditions, instead of referring them to emergency rooms, and ultimately improving timely access to primary care.

2 **Inter-professional collaborative practice models**

Advanced access enhances interprofessional collaborative practice among nurses and other primary healthcare providers by optimizing nursing practice and ensuring that both NPs and RNs work to the highest level of their skills and to their full scope of practice. This implies optimally distributing patient demand and matching patients’ needs to nurses’ competencies and expertise, and thus using them more effectively to ensure efficient workflow in the clinic.

Our results show that following implementation of AA, models of collaboration were either introduced or redesigned; these tended to be team based, maximizing the contribution of nurses, and integrate their capacities into the provision of services.

### Nurse practitioners

All NPs reported being able to practice to the full extent of their capacities in daily activities, including within the AA model. They unanimously reported, at initial and follow up interviews, having more responsibility in the AA model for addressing the needs of patients with acute illnesses and exercising a larger scope of practice. NPs emphasized their enhanced autonomous decision-making capacity and greater involvement in acute care management. They described their added value such as: becoming an active part of the first line contact in the healthcare team; increasing their availability as an entry point to care, and delivering more services for patients with common, urgent health problems and episodic illness:

"Well, I think the NP really has an added value in AA because what we see are common health problems and we have the expertise and competence and autonomy to deal with most consultations, not all of them, of course. So I think why we have so many free slots, is that within the team, I think the NP is best suited to respond to this kind of problem, like conjunctivitis, colds, urinary tract infections, injuries, wounds versus the nurse clinician who can only see patients and evaluate them." (NP1- -Interview 2)

Inter-professional collaborative practice, which was enhanced following the implementation of AA, underwent changes over time with regards to the form of collaboration and the formalization of NP’s role within this collaboration.

Various models of collaboration were observed among NPs, physicians and other team members. Some NPs shifted from a model involving mainly joint practice (pairing 1 NP with 4 to 5 physicians to follow the same panel of patients) with management of occasional referrals from other FPs /or delivering episodic care (e.g., hypertension, pregnancy follow-up) to a more formal and organized joint practice model where NPs delivered ongoing care to a well-defined patient panel. They no longer had to respond to punctual requests from other physicians in the unit after new NPs were integrated into each team of physicians.

"We have really tried to reorganize. I used to see patients from all physicians in the clinic. Because we now have additional NPs, I am for the time being more involved with the panel of patients shared with my family physician partners". (NP1-Interview 2)

Within this joint model, two types of visits were adopted, depending on the physician partner: predominantly alternating clinical visits, where the physician might see the patient once a year and the NP two or three times, and/or joint visits where NPs and physicians see patients together. Despite this change, nurses reported that physicians were planning to modify the current practice model and shift to a small team configuration in the future.

"This is what our physician directors want to focus on, to have teams composed of a NP, a nurse clinician, even a secretary, and doctors. That's the kind of the model, but it's not done yet". (RN1- -Interview 2)

Other NPs continued to operate over time within the same patient management model, such as a consultative model where the NP manages her own panel of patients and seeks assistance or advice as needed; or a team configuration model where four FPs, 1 NP, 1 secretary and 1 resident follow and provide care to an assigned panel of about 2000 patients.

“We, as NPs, chose that these would be my patients and there would be no joint follow-up. My doctors really act in a consultative way […]. So they are my own patients. When I need help with a prescription or a question, I go to them (the physicians). Maybe sometimes they come to double-check, let’s say it’s a dermatology case and they come to check the pimples. But in general, they never see my patients”. (NP4- Interview 2).

### Registered nurses

Most RNs reported that their practice was not fully optimized through attempts to operate in AA over the study period. They were not working to their full scope of practice or fully utilizing their knowledge and skills, and this was having negative effects on timely access. Data show that RNs tried to expand their responsibilities following the implementation of AA. Their experience with regards to collaborative practice reflected both efforts to change, and a lack of change.

For example, some RNs tried, over time, to shift from a non-team-based nurse pool to an integrated team model, in terms of developing small teams with four or five FPs, one RN or NP, a receptionist and a secretary. Efforts to expand their activities included joint follow-up of pregnant clients, and collective prescriptions that enabled them to adjust medications for patients with chronic illnesses. These efforts helped to free up physicians, increase timely access and enhance RNs’ relational continuity with their own patients. This change was undertaken as part of the process of implementing the AA model and optimizing healthcare provider workflow.

"Since October 2016, we have a new working model of collaboration between doctors and nurses. [ … ] There are five nurses here, so we have one nurse paired with four or five doctors, with a receptionist and a secretary. It wasn't just done for AA, it was also done to optimize the work of nurses, doctors. It was really done so nurses can help doctors liberate themselves from some patients. That's why we did it, to try to help doctors in their work" (RN3-Interview 2).

Other RNs experienced a restructuring of the interprofessional collaborative practice in which they shifted over time from being a part of a larger team, to forming small RN-secretary teamlets to handle all UFMG patients.

“As for physicians, they have their teams with their own clerical staff. Let’s say Mrs. X, a clerical staff, is paired with three physicians. She looks after minor issues. Mrs. Y, a clerical staff, has six physicians with residents. Whereas, we have a dedicated clerical staff [...], who takes care of all of us now because things have changed”; (RN5-Time 2).

However, some RNs did not manage to optimize their tasks in a lasting way. For example, two RNs, who were initially responsible for managing acute illnesses following implementation of AA, mentioned that these activities were later restricted when additional NPs were integrated into the practice. Patients were transferred from FPs to NPs, leading to a decrease in joint FP-RN follow-up. This produced uncertainty among RNs about their professional identity. RNs emphasized the need to redistribute patient management responsibilities and develop creative solutions to further delineate their professional boundaries, which were threatened within the existing team.

“All the follow-ups I used to conduct with physicians were transferred to the NPs. That was the bulk of my clientele, which I no longer have because they’re followed by a NP (...) These are not new cases, they’re the same clients who are seen by other providers. It terms of impact, I’m the one with a decrease in follow-ups. That’s it. Unless new patients come in, when we cut up the pie, we each have less” (RN2- Interview 2).

Another RN mentioned that, due to nurse understaffing, she could not be teamed with physicians to jointly manage a defined panel of patients and was still unable to work in even a limited form of AA. While she only managed patients with chronic illness, she saw her inability to assess and follow patients with acute needs as a missed opportunity to free up time for FPs.

There was a shared perception among all nurses participating in the study that actions should be taken, in the implementation of AA, to fully use RN skills, and that these would improve accessibility in the unit.

### Factors influencing changes in nursing practice in advanced access

**Fig. 1:**
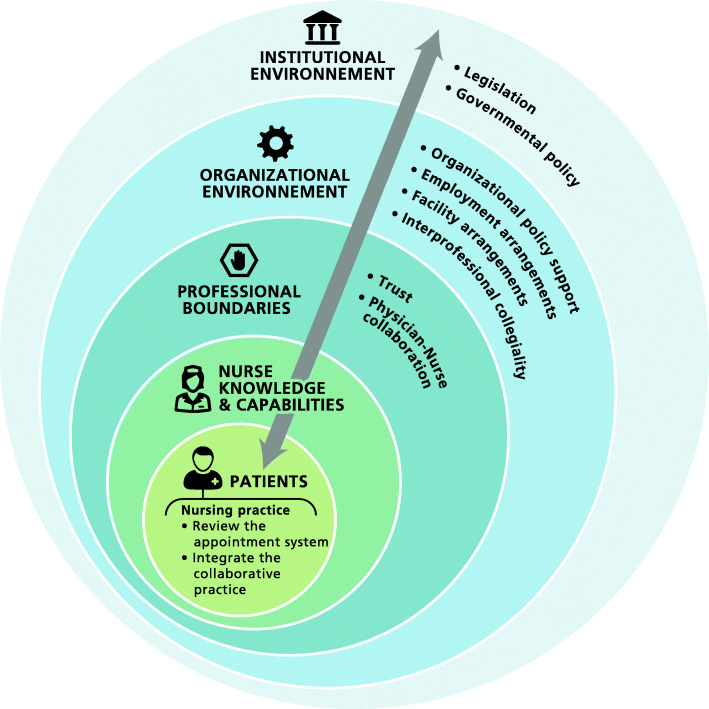
Conceptual framework to analyze factors influencing changes in nursing practices within the advanced access model

In the next stage of analysis, we identified common themes that acted as barriers or facilitators to NP and RN practice change within AA. Figure 1 illustrates the influential factors according to Niezen and Mathijssen (2014) framework on nursing practice change within advanced access.

1. Institutional environment

Policy and legislation.

A 2010 provincial policy to deploy 500 NPs in the primary healthcare setting in order to increase accessibility was mentioned by study participants as a direct trigger for changing work processes within the FMU, and indirectly for redesigning nurses’ practice within AA. This policy was seen by NPs as a facilitator of practice change. On the other hand, RNs saw it have a negative influence on their practice. One NP stressed that nurses were not a priority for decision makers at present. An RN mentioned that the deployment of a high number of NPs should have been coupled with policy to redesign RN practice and redefine professional practice boundaries.

Other institutional factors mentioned during interviews included Quebec’s Bill 20, “*An Act to enhance access to family medicine”.* The Bill pressured FPs to increase their capacity and accept new patients from the centralized waiting list for unattached patients, and was found to increase patient enrolment by FPs. It encouraged FPs to optimize RN and NP practice within a team-based care model (collaborative practice) that also featured AA.

"Personally, well, I think there's pressure to register as many patients as possible, so I think there's pressure on the clinic and on each doctor [ … ] I wasn't necessarily asked to do it, but for teamwork purposes, I also do it.

Yes, yes I'll take some [from the centralized wait list] too. For the time being, what is official is that I'm taking 10 new ones with one doctor and probably I'll register some with a few other doctors too. [ … .]. I plan to enroll more but there's no one making me feel obliged to do so, as it's a team effort. I just get on the boat".(NP3--Interview 2)

2) Organizational environment

Organizational policy support.

*Leadership*. Physician leadership was highlighted as an influential factor, whereas nurse leadership was rarely mentioned. Nurses (RNs and NPs) reported supportive leadership from physicians as an essential ingredient to implement the AA model in general, and changes in nursing practices in particular. Participants described how leadership can act as a facilitator or barrier to operating according to the AA model. Leaders who support collaborative practice among team members by enhancing or establishing teamlets, such as pairing nurses with FPs and sharing responsibility for specific patient panels, and promoting open communications channels (e.g., regular meetings) and feedback mechanisms between all team members including nurses, were seen to facilitate nurse practice change. In contrast, the absence of physician leadership to champion AA, and nurse practice optimization, was seen by some RNs and NPs as a major barrier to change. One example was expressed by an RN who was disappointed at the departure of a FP recognized as a particularly influential leader in developing processes to support her practice change within the AA model. She described how her practice regressed after the FP’s departure with respect to following up acute conditions. The absence of a champion to facilitate team engagement and effective information sharing between FPs and RNs was perceived as a barrier to RN practice in AA.

"Well, in the algorithm at the beginning that we had with Dr. X I was involved somewhere in the process, whether it was for a urinary tract infection, a patient with difficulty X, pathology Y, well, I could still see these patients.

So since she left...

I think it has fallen apart". (RN4-Interview 2)

In terms of nursing leadership, only one NP highlighted her leadership role in changing the type of appointment in her own practice and for some FPs within AA.

*Strategies to support nurses’ practice change in AA.*

There was consensus among RNs that the ‘one-size-fits-all’ training sessions offered by the Quebec family physician association (FMOQ) were not sufficient. They claimed that customized training was necessary to prepare them to change their practice within AA. A lack of exposure to the model as students and insufficient training thereafter meant they lacked a tangible understanding of how to schedule appointments and how to make patients responsible for making their own appointments, and generated fears of losing patients to follow-up. Some solutions were proposed, such as networking with and seeking assistance from other nurses in various clinical settings with experience in redesigning nursing appointment systems. Another solution seen as critical by all RNs involved formal training to prepare nurses to manage appointments and gain confidence in empowering and educating patients.

"I'd like to see how it works now. The way they work elsewhere. Sometimes, concrete information is missing. Sometimes it's an idea that remains a little abstract, then you have to see in everyday life how it's done." (RN3-Interview 2)

Additional training was not seen as an urgent need by NPs who, despite the lack of formal training upon their arrival in the unit, managed to adapt quickly to the AA system. Many reasons for this were mentioned, such as exposure to the model as students, informal training within the unit, and their involvement as “key players” in the implementation process (e.g., development of the algorithm).

Facility and employment arrangements

*The availability of human resources* (FPs, RNs, NPs) influenced nurse practice change within the AA model and acted as a barrier or facilitator depending on the setting. Staff shortages were a barrier to both RN and NP practice change. For example, one RN was unable to see patients within 24 to 48 h or undertake joint practice within AA to her full level of competency due to the insufficient number of RNs in the unit. This imposed a lot of pressure and affected her motivation. Having only one RN in the unit also impacted the NP’s practice, leading her to call for an increased number of RNs in the unit so that she might delegate more tasks to them and improve her own accessibility. Conversely, increased availability of FPs (after they dropped many offsite activities), and additional NPs had a negative impact on RN practice and restricted their ability to performing certain tasks (e.g., assessing and managing acute illness) anticipated in AA. Also, one RN reported that losing FPs to retirement led to exhaustion and lack of enthusiasm in the medical team for creating a teamlet with RNs.

A lack of nursing assistants^1^ to support RNs during patient visits, in recording vital signs, measuring weight and height, meant that RNs were performing these tasks and were not able to jointly manage clients and free up physicians to see additional patients.

“You know, I still do a lot of tasks that could be done by either a nursing assistant, or... You know, I do ear washes, I still do blood tests. If I practiced to the extent of my capacities, I could adjust diabetes medications [...] I could do alternate follow-ups, pregnancy follow-up, with the physicians, I could see babies, and free up time and appointments, and currently I don’t do it”. (RN3-Interview 2).

In the same vein, part-time positions were highlighted by two NPs as leading to a mismatch between supply and demand, and negatively affected their accessibility and the continuity of care they could provide.

"But these days it's difficult because I'm back from maternity leave, so my patients haven't seen me for over a year, and I'm also working part-time. That means I have more demand than supply here and makes AA difficult. I manage to get away with it too. I have to measure again". (NP3-Interview 2)

The knowledge and skills of clerical staff also influenced nurse practice, serving as a facilitator of AA for some RNs and a barrier for others. For example, RNs and NPs felt that a lack of a medical background and high turnover made clerical staff less likely to understand NP competencies and less able to determine patient needs and make appropriate decisions to redirect them to NPs in a timely manner.

"Well, it was definitely difficult at first because sometimes [administrative staff] would put things in my schedule that didn't work. Or precisely for urgent appointments, I am not a physician but I am part of the physicians’ team to share urgent cases between us. They had difficulty fitting me into the schedule". (NP4-Interview2)

Inter-professional collegiality

Inter-professional collegiality between NPs and RNs was perceived as a facilitator of practice change. The majority of RNs highlighted the support they received from NPs, including for conducting follow-up for patients. Even though RN activities had been transferred to NPs, some RNs mentioned that NP openness to collaborative practice helped explore new ways they might collaborate in future. NPs and RNs treated each other collegially and learned from each other. RNs talked about NP willingness to renegotiate task division based on patient needs.

"In my case, [a new NP] arrived this week, and works with the physicians with whom I work, so we're going to see how it will change my practice too. But it's probably going to be similar, so we have to look at it together anyway. The solutions often come from the people involved, so we'll have to think about that. The NP seems very open to working with the rest of us too, so we're going to look at how we can manage new clients". (RN2-Interview 2)

3) Professional boundaries

Trust, respect and an open-door policy among NPs, physicians and other team members was mentioned as a factor in improving daily practice in general as well as practice according to the AA model. There was consensus among nurses that FP commitment to meeting patient needs in a timely manner was a key facilitator to establishing and reinforcing collaborative practice between NPs and physicians.

Physician understanding of nurses’ role had an impact on collaborative practice. A clear understanding of the NP role and physician trust in NP capabilities and acceptance of their role facilitated collaborative practice, as reported by the majority of NPs.

"I always say that I'm spoiled. Here, I'm lucky to have five young physicians who understand my role, who have great confidence in me. I sometimes think they may even have too much confidence in me. [ … ] They trust me very much, but if I need them, they are always available and reachable.[ … ]. I know that if I need to, I can open the door, there's always someone there to help". (NP4-interview 2)

On the other hand, poor physician awareness of RN competencies, skills and added value was mentioned by some RNs as having a negative impact on RN practice, limiting their involvement in team-based care. Suggestions were made such as educating FPs about RN skills and competencies.

4) Individual characteristics

Knowledge and capabilities.

All NPs expressed confidence, gained through years of experience, in their capabilities to decrease demand by reducing the frequency of regular visits from healthy patients, extending the interval between return visits, and freeing up FPs by seeking their advice less often. They mentioned having greater ability to manage time, shorten appointment duration and serve more patients. One NP reported being able, with growing expertise, to eliminate case discussion slots with FPs, and replace them with appointment slots, allowing her to see more patients and be more effective.

"Yes, of course, at first, when I started working, I used to take more time for appointments, like an hour. Now I have more ability, so my appointments are shorter, I'm able to manage time better. That's what the change is all about". (NP4- Interview 2)

Only one NP, newly introduced, revealed some uncertainty in her abilities to make decisions and said she regularly had to seek FP advice, which slowed down patient flow.

Some RNs did not yet feel confident to reduce pre-booked appointments by empowering patients to call back for follow-up appointment requests, as recommended in the conceptual foundations of AA.

“When I see a patient and I say, your next diabetes follow-up, I’ll call you back in three months, I like to be able to schedule him in. But by doing that, I’m not making my patient autonomous... Should I say to my patient”, “call me back in three months”.

You're not doing it right now?

No, right now, if I see him, I'll say, "I'll call you back in three months to continue your follow-up […] That's the point, the way I work, should I make the clients more responsible and at that point say, "you call me back" so I don't fill up the two-month schedule?" (RN3-Interview 2).

5) Patient perceptions

There was a general perception among RNs that the patients’ mindset was a barrier to nurse practice change within AA. Patients were used to pre-booking appointments and preferred this traditional system where they were not responsible for calling to schedule an appointment. Participants reported that they had difficulty encouraging patients to change and that resulted in going back to the traditional system (e.g. generating a recall list). Changing well-established habits requires ongoing education of patients and regular reminders from all team members including FPs, nurses and clerical staff.

Among NPs, only one newly integrated in the unit faced patient reluctance to be seen by her rather than the physician. NP felt that patients lacked awareness of her competences and that trust was not yet established. This resulted in unused appointments slots in the NP’s schedule. This barrier was gradually being resolved with the support of the NP’s FP partner who played a role in introducing her to patients.

"The fact with AA is that when you are new in the clinic, you don't have patients because patients don't know you, so they can't call to make an appointment with you. That meant our offices were empty at first. That's been difficult and it's still difficult after six months; my slots are not often full because patients call but they don't want an appointment with me: they don't know me. [ … ] I think the best support I've had is with my partner doctors. We try to teach patients that if they have common problems or need adjustments to manage a chronic disease, they should make an appointment with me." (NP3-Interview 2)

## Discussion

This study aims to better understand how nurses - RNs and NPs - change their practice to operate within the advanced access (AA) model, and identify factors in the Quebec primary care context that influence changes in their practice. We report findings on two key principles of AA: revising the appointment system and integrating collaborative practice, which were the main focus of change among nurses.

In general, the implementation of AA resulted in a change in nursing practice in terms of revising the appointment system to increase response to urgent needs, and in terms of smoothing demand to avoid long-term scheduling. There were tangible improvements in collaborative practice and efforts were still continuing to integrate nurses’ capacities, maximize their contribution, and enable each primary care provider to use their full scope of practice. However, our data show that experiences varied in terms of practice change between the two categories of nurses. Effects of the implementation of AA on nursing practice was not equally perceived among nurses.

With regards to RNs, they reported as being the greatest losers of the AA model implementation when compared to NPs given the lack of their optimal involvement in implementing the key principles of the model.

Indeed, our results show that they made some attempt to redesign the appointment system (introducing two to three open slots per day for unscheduled care) and acknowledge that they are not really operating according to AA principles; rather, they are implementing a fairly hollow form of the model. They have limited understanding of how to organize appointments within the model, and this did not improve over the course of study. RNs were unaware that within AA, patients can book a same-day appointment with their healthcare provider for any problem (urgent, routine or preventive) [14, 17].

Among NPs, some had a better understanding and managed to redesign the appointment system based on the AA philosophy, while others adapted this key principle to their needs and the organizational setting. Our findings concur with those of Goodall et al. [14] and Pope et al. [27] that there is general uncertainty about how to operate in AA and a lack of understanding of the conceptual foundations of the model, resulting in modified versions of the appointment system. Confusion between AA appointments and urgent access was evident: all RNs and some NPs used the concepts interchangeably.

Changes in RN and NP practice within early efforts to operate according to the AA model appear ongoing and are subject to continuous adjustment. Nurses are trying to learn by trial and error, given the lack of a formal preparation and training in the model. Moreover, the need highlighted by many authors [10, 17, 27] for a period of adjustment to implement this model explains in part why nurses need more time to figure out how to adapt their scheduling system in a way that suits different practice activities.

With regards to collaborative practice, RNs made smaller efforts and were still seeking ways to extend the range of their services and to be engaged in team based-care. Designated patient panels were seen to increase service capacity, patient access and continuity of care in general and within AA. RNs were still struggling to fully use their competencies, and redefine their boundaries within an already established practice following the introduction of NPs. Their professional identity seem to be questioned and felt that they have to renegotiate their responsibilities after losing their right to perform meaningful work-tasks such as follow-up of acute conditions. NPs seemed settled and able to exercise their competencies and practice according to the AA model. They were providing independent and collaborative patient care (either consultative or in joint practice), and assuming leadership in managing patients with acute and chronic diseases.

Based on a conceptual framework that draws on Niezen & Mathijssen’s (2014) [19] network model, our data point to multiple interconnected factors at different levels – institutional, organizational, professional, individual, and patient – that influence nursing practice changes. Some factors are not specific to the AA model, given that it is embedded within unit practice and cannot be isolated from the larger system and work processes of the unit. These factors help to understand nurses’ experience in adopting and modifying components of AA. Most of the influencing factors we found were consistent with the findings of a qualitative evidence synthesis of doctor-nurse substitution strategies in primary care [27], notably with regard to organizational resources (e.g., staff shortages), leadership, clear roles, and adequate training and supervision. These were all mentioned during interviews and resonate with the literature related to implementing innovative models of care [16], reorganizing professional practice within such models [28], and optimizing nursing practice in primary care [1, 29].

Organizational environment (policy support, leadership, resources) and professional boundaries played an influential role in practice change to adopt AA model. A supportive organizational environment in terms of resources, strategies (e.g., training that meets the needs of nurses) and tools (collective prescriptions for faster processing of prescriptions) to enhance collaborative practice play an important role in creating enabling conditions for reorganizing nursing practice (RNs and NPs) and establishing team based primary care and AA. This supports findings from other studies [16, 26, 28, 30]. Training and support were seen as essential, as was networking with early adopter clinics, given the complexity of operationalizing AA to enable access management. Leadership and resources were seen as important factors, which is not surprising given that they have been found to exert a major influence on AA implementation in other studies [12, 31] and in the multiple case study [16] in which the present study is nested. Collective leadership for change through a teamwork approach, open channels of communication, and promoting a sense of egalitarianism and collective responsibility among team members are among the more powerful drivers of successfully implementing AA [16] and fully exploiting the capacity of the nursing workforce in this model of primary care. RNs should be engaged in developing and implementing AA as integral and active members of the healthcare team. Our results are supported by Beaulieu et al. (2006) [28], who showed that shared leadership with NPs in the implementation of family medicine groups (a new organizational model of practice composed of FPs in a group practice working in close collaboration with nurses) contributed to developing nurse practice quickly and intensively. Elvey and Bailey [32] also stress the need for a dialogical approach when implementing new models of care to improve primary care access, enabling staff to be engaged as active key stakeholders rather than passive participants. Engaging all healthcare providers in a horizontal-style implementation process has been recommended to manage skill mix changes [29, 33] and making optimal use of each member, including nurses, and is highlighted as a key ingredient for high-functioning teams [34].

It is important to draw attention to characteristics of the Quebec healthcare system (the institutional environment) that exert a profound influence despite not being raised frequently by study participants, and may influence the organizational environment. For example, collective/shared leadership with the nursing profession might be difficult when the delegation of tasks, distribution of cases and practice times occurs within a context where the medical group has power over the development of other professional groups and where change can disrupt fee-for-service payments [35, 36]. However, the latest legislative changes are promising in this regard: Bill 43 in October 2019 aimed to increase the power of NPs to diagnose and create treatment plans for certain conditions, as well as follow low-risk pregnancies. This change will mitigate professional struggles to some extent, help to increase access to NP services, free up FP and NP time and decrease wait times [37]. Policies and action plans to fully utilize the skills and expertise of RNs in work teams and support them in their primary care practice transformation need to be considered when implementing new primary care models (such as AA), as demonstrated in our study and recommended by others [2].

In relation to professional boundaries, our data show that NPs were considered to bring added value. On the other hand, RNs often had to defend professional boundaries and maintain their professional identity within AA. The introduction of NPs disrupted the division of tasks between the two types of nurses. RNs were struggling to fill their appointment schedules and were eager to fully utilize their competencies. This finding is echoed in many studies looking at the introduction of NPs in healthcare settings [38, 39]. Many authors highlight the need to broaden the vision and create a team dynamic, in other words focus on restructuring the entire team’s functioning and provide team-focused supports (e.g., to redesign task distribution and manage team relations) to effectively use the qualifications of all providers (including RNs) and improve organizational capacity, and ultimately accessibility [38, 39]. Indeed, maximizing RN opportunities to manage demand thro-ugh care coordination [40] and team-based care delivery models [40, 41] figures among the action-oriented priorities to improve primary care access and quality.

Our findings also highlight the role patients play in influencing nurse practice in AA, through reluctance to adopt new appointment-scheduling systems and accept to receive care from NPs and RNs. Given the growing trend to engage patients as partners in healthcare delivery improvement strategies, it would be interesting to engage well trained patients to help other patients understand and adapt to the way services are delivered within the AA model. This would also help to incorporate the preferences, values, and beliefs of patients and tailor the model to their needs [42].

In sum, this study identifies specific improvements that could be made at the institutional, organizational, professional and patient level to support primary care nursing practice transformation. Policies, professional training to transform theoretical concepts into practice, networking with nurses with established AA practices, and creating a conducive environment (leadership, resources, etc.) were suggested as ways to encourage nurse practice change within AA. Also, in making efforts to reframe professional boundaries, each professional must be working to their fullest potential, and their contributions must be aligned within this new organizational model. Finally, improvements might be designed in partnership with patients to meet their needs within the AA model.

### Strengths and limitations

One strength of this study is the use of a conceptual framework based on the AA model of Murray & Tantau (2000) [8] and the multi-layered model of Niezen & Mathijssen (2014) [19]. It provides a comprehensive understanding of the different levels (patient, individual, professional community, organization, etc.) that affect practice change within AA. The knowledge gained from our study should be taken into consideration when planning practice reorganization to implement AA in similar settings. Our qualitative approach provides an in-depth analysis of nurses’ experience with practice change to implement AA. However, generalizability of our findings is limited given that a small sample of nurses was recruited from only four healthcare settings. Another limitation is that it does not capture the views of physicians and patients regarding nurse practice in AA. Exploring their views in future will help to address subjective bias and strengthen the study findings. More research using a comparative approach or a mixed methods design is needed to understand similarities and differences between the two groups of nurses. Such studies would ideally, as mentioned earlier integrate multiple perspectives (e.g., patients, healthcare providers such as FPs, residents, clerical staff) and address bias arising from differences in the settings under study and the characteristics of nurses interviewed.

The findings of this study are particularly relevant at present for many reasons: there is a pressing need to ensure timely access to primary care services and transition to AA, which is one pillar of the patient-centered medical home; as well, there is a need to transform nursing practice in order to maximize nurses’ contribution to enhancing primary care capacity, and improving access and continuity of services in primary care. Further comprehensive evaluation is needed, specifically aimed at assessing the practice changes of nurses and other healthcare providers (e.g., social workers, psychologists) within this new organizational model in different healthcare settings. Results will help develop implementation strategies to optimize the practice of all healthcare providers within this model of primary care, and ultimately increase primary care capacity to respond to patients’ needs in a timely manner.

## Conclusions

Our study provides a first empirical foundation for future research related to changing nursing practice in AA. It suggests that healthcare organizations need to customize training to nurses’ needs and provide coaching tailored to each category of nurse, as well as critically re-examine NP and RN professional boundaries within AA, and provide the optimal professional and organizational contexts to support nurses’ practice transformation. A significant investment must be made ensuring that RNs are not marginalized, but rather involved as key actors in the implementation of AA. Thus, the study highlights the crucial need to align all team members in the current transition to AA in order to achieve the desired reductions in waiting times.

## Supplementary Information

**Additional file 1.** Interview guide.

## Data Availability

The datasets used and/or analysed during the current study are available from the corresponding author on reasonable request. The data set generated during the study will not be shared in order to respect confidentiality and not to compromise research participant privacy.

## Abbreviations

AA: Advanced access
FMOQ: Fédération des médecins omnipraticiens du Québec
FP: Family physician
UFM: University Family medicine group
MHSS: Ministry of health and social services
NP: Nurse practitioner
RN: Registered nurse

## Footnotes

1 Nursing assistants hold a diploma of professional studies. They provide nursing care usually under the direction of medical practitioners, registered nurses or other health team members. The nursing assistant contributes to the assessment of a person’s health, the execution of the care plan, and the provision of nursing care and medical treatment in order to maintain or restore the patient’s health as well as to prevent illness.
