# Supplementary material for: Changing nursing practice within primary health care innovations: the case of advanced access model
Source: BMC Nurs. 2020 Dec 2;19:115. doi: 10.1186/s12912-020-00504-z (PMC7709259; doi:10.1186/s12912-020-00504-z)
Supplement: Supplementary file 1 — Additional file 1. Interview guide. [file 12912_2020_504_MOESM1_ESM.docx]

**Interview guide**

**Date**…………………..  **Time**…………………  **Duration**…………...

1. **Socio-demographic data:**

Age:……………………………………………

Sex :…………………………………………...

Educational level:……………………………..

Years of experience:…………………………..

Position:……………………………………

1. **Characteristics of the nurse and her practice:**

- Can you tell me about your career background?
- Can you describe your current practice, i.e. your main activities as a nurse?

1. How much time do you spend caring for patients?
2. How many, what type of patients do you care for (type of illness, etc.)?
3. **Description of the progress of advanced access:**

- How long has the advanced access model been implemented in the clinic?
- How would you describe the evolution of this model since its implementation?
- How has your nursing practice been transformed since the implementation of advanced access? Please describe your experience in making this change:

1. How the planning of your time slots changed following the implementation of this model? Could you describe this change?
2. How and which changes were made to your schedule and the nature of your consultations?
3. How did your collaboration with other healthcare professionals (e.g., physicians, etc.) change? Could you describe this collaborative practice change?"
4. Which strategies you used to adjust supply to demand and respond to the patient's needs, during physicians’ absence, during your absence?
5. Which strategies were used to replace professionals, including yourself, during periods of absence?
6. **Change in the nurse’s role since the implementation of advanced access:**

- Could you describe how your role/practice has been transformed/deployed since the implementation of advanced access?

1. Would you say that you are seeing more patients (types of diseases, clientele, number of patients, etc.)?
2. Would you say that you follow up patients more autonomously compared to the period preceding the implementation of this model?
3. Are you taking on more tasks that are delegated from the physician?
4. How do you describe your collaboration with the physicians and residents of the clinic? Are you satisfied? And with others? (E.g. secretaries, others)? Are you satisfied?
5. In your opinion, what are the elements that facilitate and/or hinder this collaboration?

- Could you describe how you and the various team members have mobilized to support this change?

1. What activities and strategies were implemented to promote interdisciplinary practice (e.g., prescriptions, etc.)?
2. **Factors influencing the change/deployment of the nursing role within advanced access:**

- How do you think the context has influenced changes in nursing practice/ or the deployment of your role within advanced access? For example:

1. How did the workplace, contribute to this transformation?
2. Would you say that the professionals you work with also influenced the transformation of your role?
3. Would you consider that some of your characteristics also played a role in the role transformation/deployment?
4. Would you say that the transformation was facilitated by a leader in your clinic? Can you tell me about his or her influence on this change?
5. What other contextual factors do you think played a role?
6. In your opinion, the changes in the law will have an impact on your practice, the way you work within advanced access?

To conclude, do you want add information that was not discussed during the interview?

Thank you for your participation**!**
